# Deep Sequencing of Cancer-Related Genes Revealed *GNAS* Mutations to Be Associated with Intraductal Papillary Mucinous Neoplasms and Its Main Pancreatic Duct Dilation

**DOI:** 10.1371/journal.pone.0098718

**Published:** 2014-06-04

**Authors:** Shinichi Takano, Mitsuharu Fukasawa, Shinya Maekawa, Makoto Kadokura, Mika Miura, Hiroko Shindo, Ei Takahashi, Tadashi Sato, Nobuyuki Enomoto

**Affiliations:** First Department of Internal Medicine, Faculty of Medicine, University of Yamanashi, Chuo, Yamanashi, Japan; Deutsches Krebsforschungszentrum, Germany

## Abstract

**Background:**

To clarify the genetic mutations associated with intraductal papillary mucinous neoplasms (IPMN) and IPMN-related pancreatic tumours, we conducted cancer-related gene profiling analyses using pure pancreatic juice and resected pancreatic tissues.

**Methods:**

Pure pancreatic juice was collected from 152 patients [nine with a normal pancreas, 22 with chronic pancreatitis (CP), 39 with pancreatic ductal adenocarcinoma (PDAC), and 82 with IPMN], and resected tissues from the pancreas were collected from 48 patients (six IPMNs and 42 PDACs). The extracted DNA was amplified by multiplexed polymerase chain reaction (PCR) targeting 46 cancer-related genes containing 739 mutational hotspots. The mutations were analysed using a semiconductor-based DNA sequencer.

**Results:**

Among the 46 cancer-related genes, *KRAS* and *GNAS* mutations were most frequently detected in both PDAC and IPMN cases. In pure pancreatic juice, *GNAS* mutations were detected in 7.7% of PDAC cases and 41.5% of IPMN cases (*p*<0.001 vs. others). All PDAC cases with *GNAS* mutations (n = 3) were accompanied by IPMN. Multivariate analysis revealed that *GNAS* mutations in IPMN cases were associated with dilated main pancreatic ducts (MPD, *p* = 0.016), while no statistically independent associations with clinical variables were observed for *KRAS* mutations. In the resected pancreatic tissues, *GNAS* mutations were detected in 50% of PDAC cases concomitant with IPMN, 33.3% of PDAC cases derived from IPMN, and 66.7% of IPMN cases, while no *GNAS* mutations were detected in cases of PDAC without IPMN.

**Conclusions:**

The *GNAS* mutation was specifically found in the cases with IPMN and it was speculated that some PDACs might be influenced by the concomitant but separately-located IPMN in their pathogenic mechanism. Furthermore, the *GNAS* mutation was significantly associated with MPD dilatation in IPMN cases, suggesting its role in mucus hypersecretion.

## Introduction

Intraductal papillary mucinous neoplasm (IPMN) is a pancreatic exocrine tumour characterised by cystic dilatation of the main and/or branch pancreatic ducts; these ducts are lined with a mucin-producing atypical epithelium that often proliferates in a papillary fashion [1]–[3]. IPMN is associated with a spectrum of diseases ranging from adenoma to invasive pancreatic ductal adenocarcinoma (PDAC). PDAC may be derived from IPMN or may concomitantly develop in other regions of a pancreas in which IPMN has developed. The two IPMN-related forms of PDAC are considered to be different disease entities due to their different proximities to the IPMN in the pancreas. However, the prognosis of these IPMN-related forms of PDAC is often better than that of ordinary PDAC if early diagnosis is made [4], [5]. In contrast, the genetic characteristics of these two IPMN-related forms of PDAC and the reasons for their differing prognoses are not fully understood.

PDAC arises as a result of the accumulation of genetic and epigenetic mutations that confer a selective advantage to cancer cells [6], [7]. Mutations in *KRAS*, *CDKN2A*, *TP53*, and *SMAD4* have been frequently reported in cases of PDAC using conventional methods such as direct sequencing [8]. Recently, whole-exome analysis using next-generation sequencing has also revealed high-frequency alterations in these few genes [6], [7], [9].

In contrast, somatic oncogenic mutations in the guanine nucleotide binding protein, alpha stimulating (*GNAS*)-encoding G protein, have been recently identified in 41–66% of IPMN cases [10]–[12]. *GNAS* mutations have also been identified in several tumours of the endocrine system [13], some pituitary adenomas [14], and in McCune–Albright syndrome [15]. These mutations arise very early in the natural development of IPMN [10] and are highly specific to IPMN [11], [12], [16]. With the exception of a small percentage of pancreatic intra-epithelial neoplasias (PanINs), *GNAS* mutations have seldom been detected in most cases of PDAC or other cystic neoplasms [11], [12], [16]. However, it is unknown how the clinical presentation of IPMN may be influenced by *GNAS* mutation. Moreover, it is also unknown whether IPMN-related PDACs are associated with *GNAS* mutations.

The detection of these mutations in pancreatic juice [17]–[21] or in samples obtained by endoscopic ultrasound fine-needle aspiration (EUS-FNA) [22] aids in the diagnosis of early-stage disease. However, only a few commonly mutated genes may be analysed in small samples due to the limitations in conventional sequencing technology. To alleviate these limitations, semiconductor-based next-generation sequencing has recently been developed and enabled rapid, deep, and cost-effective sequencing of a wide range of DNA from small clinically-obtained samples [23].

In this study, semiconductor-based sequencing was employed to conduct cancer-related gene mutation analysis for pancreatic neoplasms using small tissue samples. Using pure pancreatic juice, we investigated the mutational profiling of the pancreatic neoplasms and the association of this profile with the clinical variables. In addition, using resected tissues, we compared the differences in the mutational profiling of the two types of PDACs that were related to IPMN (PDAC derived from IPMN and PDAC concomitant with IPMN).

## Materials and Methods

### Patients and samples

The pure pancreatic juices and associated clinical information were obtained from 152 cases [CP (chronic pancreatitis), n = 22; PDAC, n = 39; IPMN, n = 82; normal pancreas, n = 9] that were treated at Yamanashi University Hospital from 2000 to 2012 (Table 1). Thorough pancreatic examinations were performed in all cases. Endoscopic nasopancreatic drainage (ENPD) was performed during endoscopic retrograde cholangiopancreatography (ERCP) to obtain the pancreatic juice for cytological testing. The pure pancreatic juice was obtained and immediately stored at −80° until use. In cases with biliary disease and a normal pancreas, the ENPD was performed to avoid post-ERCP pancreatitis, and the collected pancreatic juices were classified as normal pancreatic cases in the analysis. Resected tissues were obtained at the same hospital from 2006 to 2012 from cases with IPMN (n = 6) and PDAC (n = 42), as shown in Table 2. Of the PDAC tissue specimens, 21 were macrodissected frozen specimens and 21 were microdissected formalin-fixed paraffin-embedded (FFPE) specimens, whereas six of the IPMN samples were frozen specimens. The diagnosis and classification of IPMN were based on the international consensus guidelines for the management of IPMN and mucinous cystic neoplasms of the pancreas that were established in 2006 [1]. This study was approved by the Human Ethics Review Committee of Yamanashi University Hospital. Written informed consent was obtained from all patients.

**Table 1:** Characteristics of the cases from whom the pancreatic juice was obtained.

Variable		Pancreatic juice (n = 152)			
		Normal (n = 9)	CP (n = 22)	PDAC (n = 39)	IPMN (n = 82)
Age	Median (Range)	74 (48–85)	57 (35–79)	71 (49–82)	72 (37–85)
Sex	M/F	4/5	11/11	24/15	47/35
Location	Head/Body and tail			23/16	42/40
Size (mm)	Median (Range)			22 (3–70)	
UICC Stage	0–II/III–IV			20/19	
No. of concomitant IPMN				8	
Grade of IPMN	Noninvasive/Invasive				65/17
IPMN type	Main duct/Branch				33/49
Cyst size (mm)	Median (Range)				35 (5–139)
MPD (mm)	Median (Range)				4.5 (0.5–28)

CP, chronic pancreatitis; PDAC, pancreatic ductal adenocarcinoma; IPMN, intraductal papillary mucinous neoplasm; MPD, main pancreatic duct.

**Table 2:** Characteristics of the cases from whom the pancreatic resected tissues were obtained.

		Pancreatic tissues (n = 48)			
		PDAC (n = 42)			IPMN (n = 6)
Variable		without IPMN	concomitant with IPMN	derived from IPMN	without PDAC
		(n = 26)	(n = 10)	(n = 6)	
Age	Median (Range)	67.5 (51–80)	75.5 (63–82)	75.5 (37–80)	72.5 (62–81)
Sex	M/F	14/12	6/4	4/2	5/1
Location	Head/Body and tail	15/11	8/2	1/5	2/4
Size (mm)	Median (Range)	23 (13–42)	21 (18–35)	42.5 (15–65)	
UICC Stage	0–II/III–IV	17/9	5/5	5/1	
IPMN type	Main duct/Branch		0/10	5/1	5/1
Cyst size (mm)	Median (Range)				30 (7–46)
MPD (mm)	Median (Range)				4.45 (1.2–8)

PDAC, pancreatic ductal adenocarcinoma; IPMN, intraductal papillary mucinous neoplasm; MPD, main pancreatic duct.

### DNA extraction

DNA from the resected tissues was extracted using the QIAamp DNA Mini Kit (Qiagen, Valencia, CA, USA) for the frozen specimens and QIAamp DNA FFPE Kit (Qiagen) for the FFPE specimens. DNA from the pure pancreatic juice was extracted using the DNeasy Blood Mini Kit (Qiagen) as per the manufacturer's instructions. On average, 1.4 µg and 0.3 µg of DNA were extracted from the frozen specimens and FFPE specimens, respectively, and approximately 3–4 µg of DNA were extracted from 400 µL of pure pancreatic juice.

### Preparation of amplicon libraries

The Ion AmpliSeq Cancer Panel (Life Technologies, Carlsbad, CA, USA) was used to generate target amplicon libraries, as described previously [23]. In brief, 10 ng of DNA was amplified by PCR using pre-mixed Ion AmpliSeq Cancer Primer Pools containing 190 primer pairs and the AmpliSeq HiFi Master Mix (Ion AmpliSeq Library Kit, Life Technologies). The 190 multiplexed amplicons were treated with FuPa Reagent (Life Technologies) for partial digestion of the primer sequences and phosphorylation. The amplicons were then ligated to adapters from the Ion Xpress Barcode Adapters 1–16 Kit (Life Technologies) according to the manufacturer's instructions. After ligation, the amplicons underwent nick-translation and additional library amplification by PCR to complete the linkage between the adapters and amplicons. The BioAnalyser High Sensitivity DNA Kit (Agilent, Santa Clara, CA, USA) was used to visualise the size and range and to determine the library concentrations.

### Emulsion PCR and sequencing

**Figure 1:**
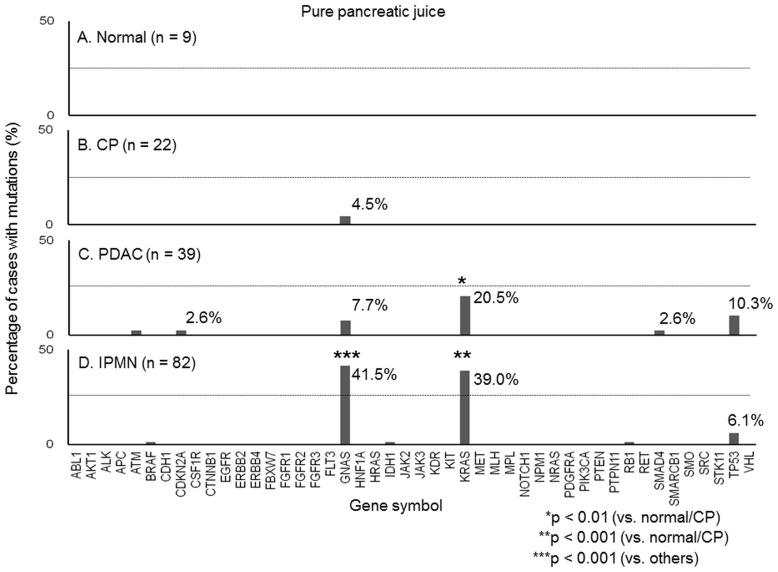
Genetic mutations in pure pancreatic juice. The y-axis of the figure represents the percentage of cases with mutations in each gene. A. No mutation was detected in the pure pancreatic juice from cases with normal pancreas tissue. B. *GNAS* mutation was detected in one case (4.5%) with CP (chronic pancreatitis) and a small cystic lesion. C. *GNAS* and *KRAS* mutations were detected in 7.7% (three of 39 cases) and 20.5% (eight of 39 cases), respectively, that had pancreatic ductal adenocarcinoma (PDAC). The *GNAS* mutation was detected in all cases with intraductal papillary mucinous neoplasm (IPMN, n = 3). D. *GNAS* and *KRAS* mutations were detected in 41.5% (34 of 82 cases) and 39.0% (32 of 82 cases), respectively, with IPMN.

**Figure 2:**
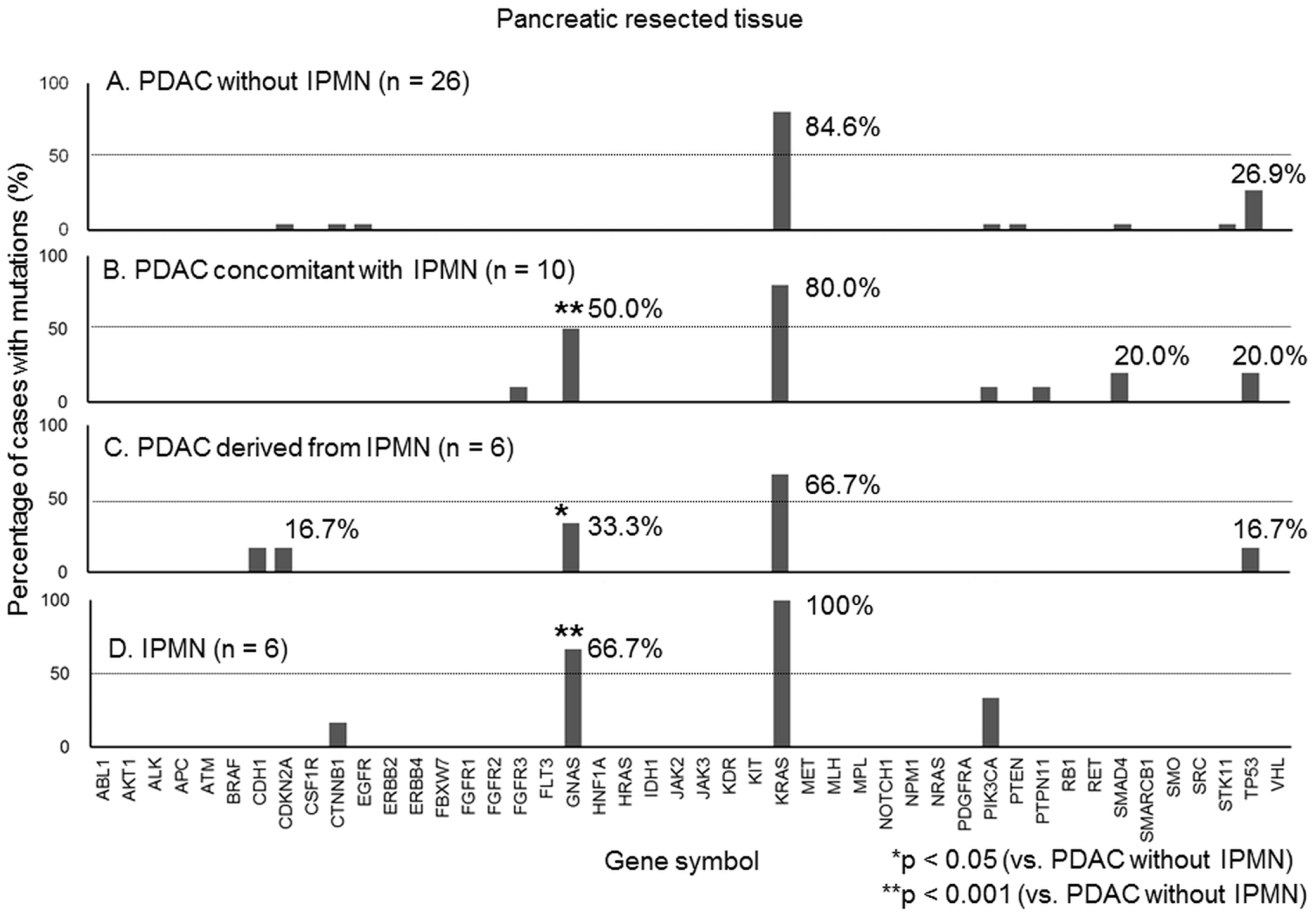
Genetic mutations in resected tissues. The y-axis of the figure represents the percentage of cases with mutations in each gene. A. In the resected tissue, the *KRAS* mutation was detected in 84.6% (22 of 26 cases) of the pancreatic ductal adenocarcinoma (PDAC) cases that did not have intraductal papillary mucinous neoplasm (IPMN). B. *KRAS* and *GNAS* mutations were detected in 80% (eight of 10 cases) and 50% (five of 10 cases), respectively, of PDAC cases concomitant with IPMN. C. *KRAS* and *GNAS* mutations were detected in 66.7% (four of 6 cases) and 33.3% (two of 6 cases), respectively, of PDAC cases derived from IPMN. D. *KRAS* and *GNAS* mutations were detected in 100% (n = 6) and 66.7% (four of 6 cases), respectively, of noninvasive IPMN cases.

Multiplexed barcoded libraries were amplified by emulsion PCR on Ion Sphere particles (ISPs) using the Ion One Touch 200 Template Kit v2 (Life Technologies) as per the manufacturer's instructions. After the template ISPs were recovered from the emulsion, the positive template ISPs were biotinylated during the emulsion process and enriched with Dynabeads MyOne Streptavidin C1 beads (Life Technologies). Sequencing was performed on a Personal Genome Machine Sequencer (Life Technologies) using the Ion PGM 200 Sequencing Kit (Life Technologies) according to the manufacturer's instructions. Since the DNA-mutated tumour cells might exist as minor populations in the samples because of the small proportions among the whole tumour tissues and/or the contamination with non-tumour cells, deep sequencing analysis was conducted in this study to detect mutations at a rate as low as 1%. Torrent Suite v2.2 software (Life Technologies) was used to parse the barcoded reads, to align reads to the reference genome, and to run metrics, including chip-loading efficiency and total read counts and quality. Variants were identified with Variant Caller v2.0 software (Life Technologies). The quality value of the targeted base was set at 21, which is equal to 0.79% of the probability for error in mutation detection. The threshold of the mutation detection ratio was set at ≥1%. The Ion AmpliSeq Cancer Panel (Life Technologies), which was used in library amplification, targets 739 mutation sites of 46 cancer-related genes that were reported in the Catalogue of Somatic Mutations in Cancer (COSMIC; hotspot mutations) [24]; these detected hotspot mutations were analysed in combination with the clinical variables. The 46 cancer-related genes are listed on the horizontal axis of the graph in Figure 1 and Figure 2.

### Statistical analysis

The associations between the gene mutations and clinical variables were evaluated using Fisher's exact test or the χ^2^ test and variables with a *p* value<0.2 were included in the multivariate analysis. Continuous data such as cyst size and presence of mural nodules were categorized according to receiver operating characteristic (ROC) analysis (data not shown). For the multivariate analysis, a multiple logistic regression model was used, and a *p*-value<0.05 was considered to be significant.

## Results

### Deep sequence analysis of 46 cancer-related genes in pure pancreatic juice

**Figure 3:**
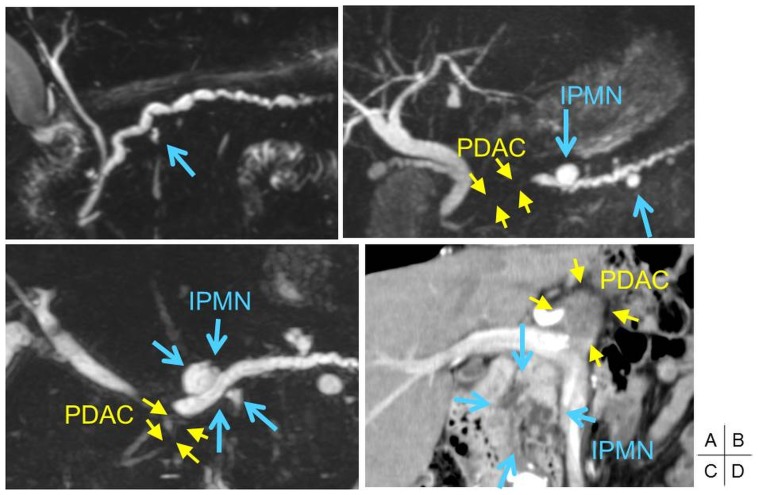
Radiological imaging of pancreatic ductal adenocarcinoma (PDAC) concomitant with intraductal papillary mucinous neoplasm (IPMN). A. A case of CP with a small cyst (blue arrow) that had the *GNAS* mutation (Fig. 1B) in pure pancreatic juice on magnetic resonance cholangiopancreatography (MRCP) imaging. B–D. PDAC with *GNAS* mutations distinct from concomitant IPMN. In cases 30 (B) and 31 (C), MRCP imaging revealed stenosis in the MPD in the pancreatic head near the PDAC (yellow arrowhead). The IPMN was located in the pancreatic body (blue arrow). Computed tomographic imaging in case 28 (D) revealed that the PDAC was located in the pancreatic body (yellow arrowhead), whereas the IPMN was located in the pancreatic head (blue arrow). A list of cases B–D is shown in Table 4.

The cancer-related mutations in the 46 genes were analysed in the pure pancreatic juice of the 152 cases, with an average of 2,114 reads per amplicon. The extracted variants with the list of altered genes, variant frequency, read depth, and corresponding COSMIC IDs from the deep-sequencing data of each case have been attached as supporting information to this paper as Microsoft Excel-formatted files (File S1–S4). No mutation was detected in the pancreatic juice of the nine cases with normal pancreatic tissue (Fig. 1A). A *GNAS* mutation was detected in one case with CP that was accompanied by a small cyst (5 mm); this may have been representative of an early IPMN lesion (Fig. 1B and Fig. 3A). *KRAS*, *GNAS*, and *TP53* mutations were detected in both PDAC and IPMN cases (Fig. 1). *KRAS* mutations were detected in 20.5% of PDAC cases (*p* = 0.0074 vs. normal and CP) and 39.0% of IPMN cases (*p*<0.001 vs. normal and CP). *GNAS* mutations were detected in 7.7% of PDAC cases and 41.5% of IPMN cases (*p*<0.001 vs. others). IPMN was present in all PDAC cases that contained the *GNAS* mutation (n = 3).

Since our analysis demonstrated that the *GNAS* mutation was specific to IPMN, the associations between the selected clinical variables and *GNAS* status in the 82 IPMN cases were statistically analysed to clarify the clinical relevance and importance of the *GNAS* mutation in the pancreatic juice. The univariate analysis revealed an association between the *GNAS* mutation and the IPMN type (main duct type) and amount of dilatation (≥6 mm) in the MPD (Table 3). In the multivariate analysis, only MPD dilatation was independently associated with *GNAS* mutation (Table 3). On the other hand, the *KRAS* mutation was associated with the location (pancreas body and tail, *p* = 0.044) and amount of dilatation (≥6 mm) in the MPD (*p* = 0.01) by the univariate analysis; however, there was no independent clinical association with this mutation after the multivariate analysis (data not shown).

**Table 3:** Results of univariate and multivariate analyses of *GNAS* status in the pancreatic juice of cases with IPMN.

			*GNAS* status			Univariate		Multivariate	
		Total	WT	Mutation		*p* value		Odd's ratio (95% CI)	*p* value
		n	n	n	(%)				
Age	≤65	24	14	10	(41.7)	0.981			
	>65	58	34	24	(41.4)				
Sex	M	47	28	19	(40.4)	0.825			
	F	35	20	15	(42.9)				
Location	Ph	34	20	14	(41.2)	0.970			
	Pbt	24	14	10	(41.7)				
Tumours	Benign	45	29	16	(35.6)	0.231			
	Malignant	37	19	18	(48.6)				
IPMN type	Main duct	33	15	18	(54.5)	0.048 ^†^		1.181 (0.384–3.629)	0.772
	Branch	49	33	16	(32.7)				
Cyst size	≤30 mm	26	13	13	(50.0)	0.166		0.374 (0.121–1.150)	0.086
	>30 mm	45	30	15	(33.3)				
Mural nodule	≤6 mm	55	32	23	(41.8)	0.926			
	>6 mm	27	16	11	(40.7)				
MPD	≥6 mm	26	10	16	(61.5)	0.010 ^†^		4.374 (1.324–14.451)	0.016 ^†^
	<6 mm	46	32	14	(30.4)				
Number of cyst	<3	58	30	28	(48.3)	0.052		0.364 (0.105–1.266)	0.112
	≥3	24	18	6	(25.0)				

WT, wild type; IPMN, intraductal papillary mucinous neoplasm;

MPD, main pancreatic duct (normal MPD diameter ≤2 mm);

Ph, pancreas head; Pbt, pancreas body and tail; ^†^ *p*<0.05.

### Deep sequence analysis of 46 cancer-related genes in resected pancreatic tumour tissues

After we found the *GNAS* mutation in cases that had PDAC concomitant with IPMN as well as in cases that had only IPMN from the analysis of the pancreatic juice, we next sequenced tissue samples to determine the possible mutational patterns in IPMN, PDAC derived from IPMN, PDAC that was with concomitant IPMN, and PDAC alone. The sequencing was performed in 48 resected pancreatic tumours (PDAC without IPMN, n = 26; PDAC concomitant with IPMN, n = 10; PDAC derived from IPMN, n = 6; noninvasive IPMN, n = 6; Fig. 2). The average number of reads per amplicon was 2,582. The extracted variants with the list of altered genes, variant frequency, read depth, and corresponding COSMIC ID from the deep-sequencing data of each case have been attached to this paper as supporting information in Microsoft Excel-formatted files (File S5–S8). *KRAS* mutations were detected in 84.6% of PDAC cases without IPMN, 80% of PDAC cases that were concomitant with IPMN, 66.7% of PDAC cases that were derived from IPMN, and 100% of IPMN cases. *GNAS* mutations were detected in 50% of PDAC cases that were concomitant with IPMN (*p* = 0.0007 vs. PDAC without IPMN), 33.3% of PDAC cases that were derived from IPMN (*p* = 0.03 vs. PDAC without IPMN), and 66.7% of IPMN cases (*p* = 0.0004 vs. PDAC without IPMN), while no *GNAS* mutations were detected in cases of PDAC without IPMN. *GNAS* mutations were thus only detected in IPMN and IPMN-associated PDAC (PDAC concomitant with IPMN and PDAC derived from IPMN). A detailed list of gene mutations is provided in Table 4. Thus, similar characteristics in gene mutations were observed for these three disease categories (Table 4).

**Table 4:** Cancer-related gene profiling in resected pancreatic tumours.

Case	Age	Sex		Diagnosis		*GNAS*			*KRAS*		
					Mutation	VF (%)	Coverage	Mutation	VF (%)	Coverage	
1	62	M			WT			G12D	23.0	634	
2	73	M			WT			G12R	16.4	691	
3	68	M			WT			G12D	12.2	809	
4	63	F			WT			G12V	6.9	800	
5	62	F			WT			G12V	8.8	2567	
6	77	M			WT			G12D	11.8	1441	
7	51	F			WT			G12D	12.0	1731	
8	72	M			WT			G12D	14.5	689	
9	77	F			WT			G12V	20.3	723	
10	65	F			WT			G12D	20.5	985	
11	67	M			WT			G12V	34.8	863	
12	60	M			WT			A146T	1.21	990	†
13	53	M		without IPMN	WT			G12D	19.24	2355	†
14	67	M			WT			G12D	16.13	595	†
15	78	M			WT			G12D	1.87	268	†
6	73	F			WT			G12R	28.22	1244	†
17	57	F			WT			G12R	55.63	6899	†
18	52	M			WT			G12V	13.59	2126	†
19	72	F			WT			G12V	4.92	488	†
20	71	F			WT			G12V	32.44	4263	†
21	61	M	PDAC		WT			G12V	30.55	4458	†
22	78	M			WT			Q61H	23.27	1985	†
23	78	F			WT			WT			†
24	80	F			WT			WT			†
25	71	F			WT			WT			
26	67	M			WT			WT			
27	82	F			R201C	5.5	2321	G12D	6.5	1364	
28	78	M			R201H	13.8	4075	G12D	13.2	2058	†
29	72	M			R201H	5.7	2440	G12D	14.4	2145	
30	69	M		with IPMN	R201C	1.0	2235	WT			†
31	80	M		(PDAC concomitant	R201H	5.8	925	WT			
32	73	M		with IPMN)	WT			G12V	25.4	688	†
33	78	F			WT			G12V	6.5	851	†
34	73	M			WT			G12V	1.9	1134	†
35	63	F			WT			G12D	11.3	1185	
36	79	F			WT			G12D	14.9	2563	†
37	72	M			R201C	10.4	501	G12V	10.5	467	
38	79	M			R201C	11.8	2154	G12D	16.2	1292	†
39	79	F		PDAC derived from	WT			Q61H	10.5	873	
40	80	F		IPMN	WT			G12D	16.9	2330	†
41	69	M			WT			WT			
42	37	M			WT			WT			
43	81	M			R201H	46.6	1302	Q61H	48.0	1628	
44	69	M			R201C	49.6	1215	G12D	42.9	815	
45	78	F	IPMN	without PDAC	WT			G12V	39.3	751	
46	62	M			R201H	39.4	1089	G12V	38.3	650	
47	67	M			R201H	16.1	1051	G12D	16.2	1183	
48	76	M			WT			G12D	7.0	512	

VF, Variant frequency; WT, wild type;

PDAC, pancreatic ductal adenocarcinoma; IPMN, intraductal papillary mucinous neoplasm.

† DNA from formalin-fixed, paraffin-embedded samples.

### Radiological images of PDAC concomitant with IPMN

The radiological images for cases 28, 30, and 31 in Table 4 are shown in Figure 3 as representative images that demonstrated PDAC concomitant with IPMN. In these cases, the *GNAS* mutations were detected in PDAC and the concomitant IPMN was located in a separate pancreatic region. For example, pancreatic duct strictures as a result of PDAC were evident in the pancreatic head in case 30 (Fig. 3B) and case 31 (Fig. 3C), while the IPMN was visible in the pancreatic body. In case 28 (Fig. 3D), a low-density mass lesion of PDAC and the multiple cystic lesions of IPMN were apparently separated in the pancreas.

### Association of mutational patterns between pancreatic juice and resected pancreatic tumour tissues

Some genes such as *ATM*, *BRAF*, *IDH1*, and *RB1* were detected only in the pancreatic juice (Fig. 1) but not in the resected tissues (Fig. 2). In contrast, the percentage of cases with mutations of *KRAS* or *GNAS* was higher in the resected tissues than in the pancreatic juice (Fig. 1, 2). In order to investigate if the mutational profiles of the pancreatic juice properly represented those of the pancreatic tumours, the mutational profiles between the pancreatic juice and resected pancreatic tumour tissues were compared in 21 cases for which both samples were available. Among the *KRAS* (n = 17) and *GNAS* (n = 7) mutations found in the 21 resected pancreatic tumour tissues, the *KRAS* and *GNAS* mutations were detected in the pancreatic juice in 41.2% (7/17) and 71.4% (5/7) of cases, respectively (Table 5), indicating that the sensitivity of pancreatic juice for the detection of mutations of pancreatic tumours may reach approximately 50%. If we look at the specifics among the *KRAS* (n = 11) and *GNAS* (n = 4) mutations found in the resected pancreatic tissues of the15 PDAC cases, *KRAS* and *GNAS* mutations were detected in the pancreatic juice of 27.3% (3/11) and 50% (2/4) of cases, respectively. Among the *KRAS* (n = 6) and *GNAS* (n = 3) mutations found in the resected pancreatic tissues of the six IPMN cases, *KRAS* and *GNAS* mutations were detected in the pancreatic juice in 66.7% (4/6) and 100% (3/3) of the cases, respectively. The percentage of cases with mutations were higher in IPMN than in PDAC; therefore, we thought that the mutations detected in the pancreatic juice could be alternative way to measure the mutations in those from tissues at least in the cases with IPMN, according to this analysis. As a result, we analysed the association between the clinical variables and *GNAS* status with the pancreatic juice in the cases with IPMN.

**Table 5:** Comparison of detected mutations between the pancreatic juice and resected tissue.

						*GNAS*		*KRAS*	
Case	Age	Sex	Diagnosis	IPMN-Type		Mutation	VF (%)	Mutation	VF (%)
1	73	M				WT		G12R	16.35
						WT		WT	
2	62	F				WT		G12V	8.77
			PDAC without			WT		WT	
3	68	M	IPMN			WT		G12D	12.24
						WT		WT	
4	62	M				WT		WT	
						WT		G12D	23.03
5	82	F		Branch		R201C	5.47	G12D	6.45
						R201C	12.77	WT	
6	73	M		Branch		WT		G12V	1.94
						WT		G12V	31.79
7	78	F		Branch		WT		G12V	6.46
						WT		G12V	12.79
8	72	M	PDAC	Branch		R201H	5.7	G12D	14.36
			concomitant			WT		WT	
9	63	F	with IPMN	Branch		WT		G12D	11.31
						WT		WT	
10	73	M		Branch		WT		G12V	25.44
						WT		WT	
11	80	F		Branch		WT		G12D	16.91
						WT		WT	
12	80	M		Branch		R201H	5.84	WT	
						WT		WT	
13	72	M		Main duct		R201C	10.38	G12V	10.49
						R201C	27.82	G12V	21.62
14	69	M	PDAC derived	Main duct		WT		WT	
			from IPMN			R201C	8.9	G12V	17.17
15	37	M		Branch		WT		WT	
						WT		WT	
16	67	M		Main duct		R201H	10.53	G12D	16.2
						R201H	16.08	G12D	16.23
17	81	M		Main duct		R201H	46.62	Q61H	48.03
						R201H	14.39	Q61H	17.46
18	76	M		Main duct		WT		G12D	7.03
			IPMN			WT		G12D	17.28
19	78	F	(Noninvasive)	Main duct		WT		G12V	39.28
						WT		G12V	25.22
20	79	F		Main duct		WT		Q61H	10.54
						WT		WT	
21	62	M		Branch		R201H	39.39	G12V	38.31
						R201H	9.38	G12D	5.92
No. of detected mutations in tissue samples						7		17	
No. of pancreatic juice mutations identical to tissue samples						5		7	
Concordance rate of mutation					(%)	71.4		41.2	

Upper lines of each case indicate the results for tissue samples and lower lines indicate the results for pancreatic juice.

VF, Variant frequency; WT, wild type;

PDAC, pancreatic ductal adenocarcinoma; IPMN, intraductal papillary mucinous neoplasm.

## Discussion

In this study, semiconductor-based DNA sequencing was performed in clinically-obtained samples of pure pancreatic juice and resected pancreatic tissue, focusing on IPMN and IPMN-associated tumours. The analysis of the pancreatic juice revealed that the *GNAS* mutation was specific to IPMN and that the *GNAS* mutation was specifically associated with IPMN with MPD dilatation. The analysis of the resected tissues with regard to the *GNAS* mutational status demonstrated that IPMN-associated PDAC (both PDAC concomitant with IPMN and PDAC derived from IPMN) shared similar mutational characteristics and that these characteristics differed from those of ordinary PDAC.

Among the 46 cancer-related genes sequenced in our study, *KRAS* and *GNAS* mutations were detected with high frequency while others including *TP53*, *SMAD4*, and *CDKN2A* mutations were detected in only a few cases as stated before. Since there have been several previous reports that have analysed *KRAS* mutations in PDAC or IPMN, we focused our analysis on the *GNAS* mutation. Recent advances in next-generation sequencing have revealed detailed genetic alterations in cases with PDAC [6], [7], [9] and IPMN [12], [16]. Jones et al [7], Biankin et al [9], and Wang et al [6] conducted whole-exome sequencing analysis of pancreatic PDAC and revealed that *KRAS*, *TP53*, *SMAD4,* and *CDKN2A* were the most frequently altered genes, as reported previously [8]. Of these four genes, mutations for the *TP53*, *SMAD4*, and *CDKN2A* genes were detected in only a few cases with PDAC in our study. Though homozygous deletion has been reported in the *SMAD4* and *CDKN2A* genes in previous studies [8], precise identification of the homozygous deletion in clinical samples has been difficult because of DNA contamination from normal cells. However, successful detection of homozygous DNA deletion was possible for these genes in the analysis of pure pancreatic cancer cell lines (data not shown). Because only the 73 *TP53* hot spot mutations from among more than 1000 mutation sites listed in the COSMIC database were targeted by the Ion AmpliSeq Cancer Primer Pools [24] and, moreover, low tumour-cellularity specimens were used, the *TP53* alterations may have been underestimated in our study. These gene alterations could have been detected by other methods, such as immunohistochemistry or copy-number variation analysis. It seemed odd that some mutations as *ATM, BRAF, IDH1*, and *RB1* were only detected in the pancreatic juice and not in the resected specimens. However, since every tissue used for the analysis was only a part of the whole tumour, it was possible that the pancreatic juice might be more representative of mutations from the entire tumour in certain conditions.

Wu et al [16] and Furukawa et al [12] performed whole-exome sequencing of IPMN from resected tissues and found that *KRAS*, *GNAS*, and *RNF43* were frequently mutated genes. In this study, the *KRAS* and *GNAS* mutations were also detected in the pancreatic juice and resected tissues from the cases with IPMN. *GNAS* mutations have been reported in several neoplasms in the endocrine system [13], [24], some pituitary adenomas [14], and in McCune–Albright syndrome [15]. *GNAS* mutations have also been previously reported in IPMN [11], [12] cases that had pancreatic cysts [10], [12], [16] with a detection rate of 41–66% from resected tissues, pancreatic cyst fluids, or secretin-stimulated pancreatic juice collected from the duodenum. More recently, it has been reported that intraductal papillary neoplasms of the bile duct [25], [26], pyloric gland adenoma of the stomach and duodenum [27], and low grade appendiceal mucinous neoplasms [28] resembled IPMN histologically and that these neoplasms had a high frequency of *GNAS* mutations. *GNAS* encodes the α-subunit of a stimulatory G-protein (Gαs) and a mutation in *GNAS* causes the constitutive activation of adenylyl cyclase and an elevated cAMP level [29], [30]. Although the roles/functions of the *GNAS* mutations in IPMN or PDAC have not been elucidated, these mutations may be associated more with tumour initiation than with tumour progression because the mutation has also been observed in low-grade tumours [10], [12]. In our study, *GNAS* mutations were detected in 41.5% of the pure pancreatic juice and in 66.7% of the resected tissue specimens from cases with IPMN; these mutation rates were nearly equal to those in previous reports. In contrast, *KRAS* mutations have been reported in resected tissues from both IPMN and PDAC, with respective detection rate ranges of 48–81% [12], [16] and 78–100% [7], [9], [12], indicating that *KRAS* mutations can be associated with both diseases; however, the mutation rate has been somewhat higher in PDAC. The *KRAS* mutation detection rates in our study were equivalent to those studies of the resected tissues in both the IPMN and PDAC cases.

*GNAS* mutations were associated with MPD dilatation in IPMN cases through the multivariate analysis. Kanda et al reported an independent association of *GNAS* mutations with the development of multiple cysts in cases with pancreatic cysts and Marco et al reported that IPMN with an intestinal phenotype has always been associated with *GNAS* mutations [10], [31]. Our data confirmed the specificity of *GNAS* mutations to IPMN and suggested a functional role of *GNAS* mutations in the formation of IPMN. In particular, since MPD dilatation without obstruction in IPMN has been generally considered to be the result of mucus hypersecretion, *GNAS* mutations may have a role in the upregulation of mucus hypersecretion. Actually, it has been recently reported that the introduction of the *GNAS* mutation in cultured cells results in the upregulation of mucin genes, supporting our hypothesis [32]. A recent study reporting the high frequency of the *GNAS* mutation, especially in intestinal type IPMN that has conspicuous mucus production, supported our results as well [31]. Further studies of a possible causal relationship between *GNAS* mutations and MPD dilatation are warranted.

**Figure 4:**
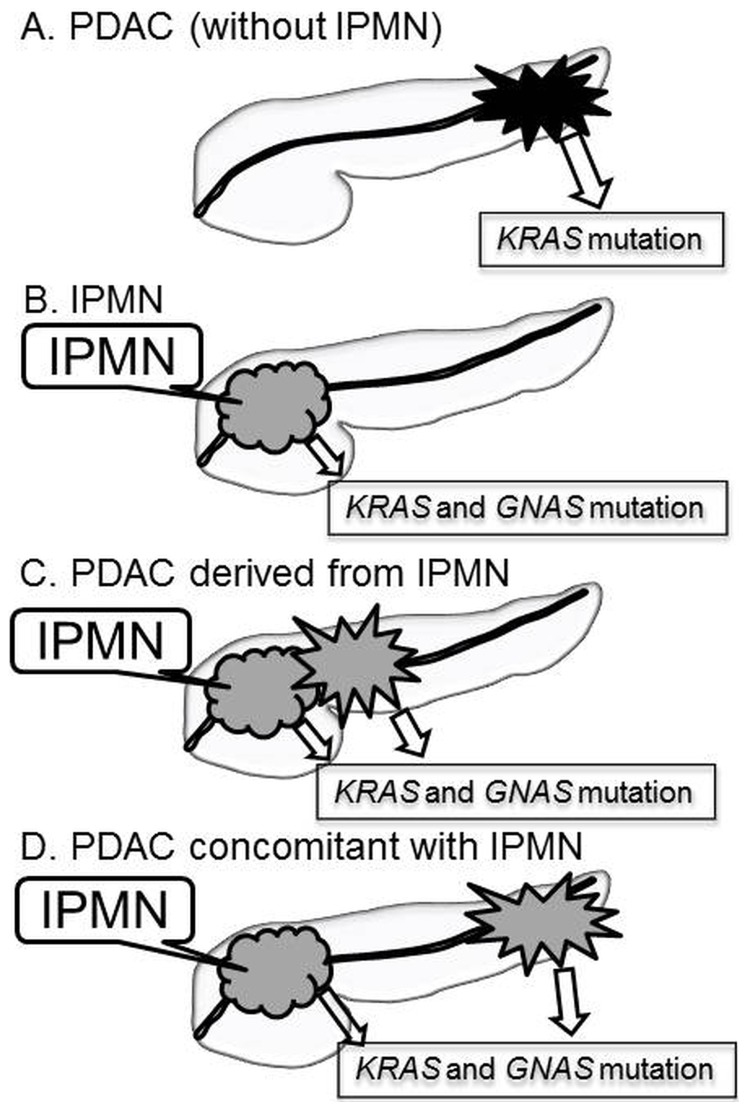
Schema of pancreatic ductal adenocarcinoma (PDAC) with associated intraductal papillary mucinous neoplasm (IPMN). A. The *KRAS* mutation was detected in ordinary PDAC without IPMN. B. *KRAS* and *GNAS* mutations were detected in IPMN. C. *KRAS* and *GNAS* mutations were detected in PDAC derived from primary IPMN. D. *KRAS* and *GNAS* mutations were detected not only in IPMN but also in PDAC that developed separately from IPMN.

Similar mutation patterns of cancer-related genes were observed between the PDAC that was concomitant with IPMN and the PDAC derived from IPMN in this study. In previous studies, *GNAS* mutations have not been detected in ordinary PDAC with a few exceptions [12], [33]. Conventionally, these tumours have been classified into three groups: ordinary PDAC, PDAC concomitant with IPMN and PDAC derived from IPMN [4], [5]. A schematic classification of PDAC in terms of the relationship with IPMN is provided in Figure 4. In ordinary PDAC, no IPMN is identified in the pancreas (Fig. 4A). PDAC derived from IPMN is an invasive cancer in which a histological transition from IPMN to PDAC may be observed (Fig. 4C) [4]. On the other hand, PDAC concomitant with IPMN is a ductal adenocarcinoma that is considered to be a different disease entity from the PDAC derived from IPMN (Fig. 4D); the PDAC lesions are separate and distinct from the concomitant IPMN lesions in the pancreas on radiological imaging and histological examination. An annual incidence of an independent PDAC development of 0.8–4.1% has been reported in cases with IPMN during follow-up [5], [34]–[36]. Although PDAC concomitant with IPMN may develop independently of IPMN, its biological behaviour resembles that of PDAC derived from IPMN because its outcome is more favourable compared with the outcomes of ordinary PDAC. Although another report studying *GNAS* status in PDAC concomitant with IPMN showed no mutation in six PDAC concomitant with IPMN cases, their detection sensitivity may have been lower than expected because they only detected *GNAS* mutations in 1 of 6 IPMN cases [37]. From the mutational pattern of *GNAS* observed in this study, we speculated that PDAC concomitant with IPMN and PDAC derived from IPMN may share some similar molecular mechanisms with the pathogenesis of PDAC.

Our research proved that cancer-related gene profiling by semiconductor-based sequencing is possible using small clinical samples. On the other hand, the detection sensitivity was somewhat lower in our analysis partly because of insufficient sequencing depth for each gene for a large number of genes targeted in a single analysis. Therefore, in future studies, the selection of more appropriate target genes and technical refinement of the sequence coverage may improve both sensitivity and specificity. It may be possible in the future to qualitatively diagnose tumours and to individually treat through the mutational analysis of small clinical samples such as aspirated tissues by EUS-FNA or microdissected tissues from formalin-fixed, paraffin-embedded samples.

In summary, cancer-related gene profiling was performed in this study by semiconductor-based sequencing of a small sample. The data identified in this study revealed that the *GNAS* mutation was specifically detected in IPMN and IPMN-associated PDAC and was associated with MPD dilatation. These findings may facilitate qualitative and quantitative diagnosis of pancreatic tumours and personalised therapy in the future.

## Supporting Information

File S1 **Deep-sequencing data for nine pure pancreatic juice in normal pancreas.**
(XLSX)

File S2 **Deep-sequencing data for 22 pure pancreatic juice in chronic pancreatitis.**
(XLSX)

File S3 **Deep-sequencing data for 39 pure pancreatic juice in pancreatic ductal adenocarcinoma (PDAC).**
(XLSX)

File S4 **Deep-sequencing data for 82 pure pancreatic juice in intraductal papillary mucinous neoplasms (IPMN).**
(XLSX)

File S5 **Deep-sequencing data for 26 resected tissues from PDAC without IPMN.**
(XLSX)

File S6 **Deep-sequencing data for 10 resected tissues from PDAC concomitant with IPMN.**
(XLSX)

File S7 **Deep-sequencing data for six resected tissues from PDAC derived from IPMN.**
(XLSX)

File S8 **Deep-sequencing data for six resected tissues from IPMN.**
(XLSX)
